# Altered resting activity patterns and connectivity in individuals with complex regional pain syndrome

**DOI:** 10.1002/hbm.25087

**Published:** 2020-06-08

**Authors:** Flavia Di Pietro, Barbara Lee, Luke A. Henderson

**Affiliations:** ^1^ Department of Anatomy and Histology Brain and Mind Centre, University of Sydney Sydney Australia; ^2^ School of Pharmacy and Biomedical Sciences Faculty of Health Sciences, Curtin University Perth Australia

**Keywords:** chronic pain, CRPS, infra‐slow oscillations, primary somatosensory cortex, resting state fMRI, tactile acuity, thalamus

## Abstract

Complex regional pain syndrome (CRPS) is a chronic neuropathic pain disorder that typically occurs in the limbs, usually the upper limb. CRPS usually develops from a peripheral event but its maintenance relies on changes within the central nervous system. While functional abnormalities in the thalamus and primary somatosensory cortex (S1) of the brain are some of the most consistently reported brain findings in CRPS, the mechanisms are yet to be explored in full, not least of all how these two regions interact and how they might relate to clinical deficits, such as the commonly reported poor tactile acuity in this condition. This study recruited 15 upper‐limb CRPS subjects and 30 healthy controls and used functional magnetic resonance imaging (fMRI) to investigate infra‐slow oscillations (ISOs) in critical pain regions of the brain in CRPS. As hypothesised, we found CRPS was associated with increases in resting signal intensity ISOs (0.03–0.06 Hz) in the thalamus contralateral to the painful limb in CRPS subjects. Interestingly, there was no such difference between groups in S1, however CRPS subjects displayed stronger thalamo‐S1 functional connectivity than controls, and this was related to pain. As predicted, CRPS subjects displayed poor tactile acuity on the painful limb which, interestingly, was also related to thalamo‐S1 functional connectivity strength. Our findings provide novel evidence of altered patterns of resting activity and connectivity in CRPS which may underlie altered thalamocortical loop dynamics and the constant perception of pain.

## Graphical abstract

Individuals with complex regional pain syndrome (CRPS) displayed increased infra‐slow oscillations (0.03–0.06 Hz) in the thalamus contralateral to the painful limb as well as stronger thalamo‐S1 functional connectivity than controls. CRPS subjects displayed poor tactile acuity on the painful limb which was also related to thalamo‐S1 functional connectivity strength. Our findings provide novel evidence of altered patterns of resting activity and connectivity in CRPS which may underlie altered thalamocortical loops dynamics and the constant perception of pain.

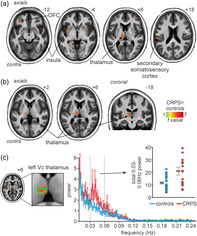

## 1 INTRODUCTION

Complex regional pain syndrome (CRPS) is a chronic pain disorder characterised by spontaneous or regionally evoked pain and other signs and symptoms typically affecting the distal extremities, particularly the upper limbs (Marinus et al., 2011). Whilst CRPS usually develops after a peripheral event, it is likely maintained by changes in the central nervous system (Marinus et al., 2011; van Rijn et al., 2011). There are several lines of evidence of central nervous system involvement. These include the observation that the pain often spreads in a non‐dermatome fashion (van Rijn et al., 2011), that pain intensity of the initial injury, but not injury severity, predicts the development of CRPS (Moseley et al., 2014), as well as the presence of perceptual deficits including altered two‐point discrimination ability (Galer & Jensen, 1999a; McCabe, Haigh, Halligan, & Blake, 2003; Moseley, 2005). Despite evidence of higher changes, the neural mechanisms responsible for CRPS remain unknown.

Multiple human brain imaging studies have shown that chronic neuropathic pain is not associated with ongoing increases in activity in the classic ‘pain pathways’, but instead a reduction in thalamic blood flow and functional ‘cortical reorganisation’ in the primary somatosensory cortex (S1) are arguably the most consistent findings (Di Pietro et al., 2013; Hsieh, Belfrage, Stone‐Elander, Hansson, & Ingvar, 1995; Iadarola et al., 1995; Youssef et al., 2014). Furthermore, in addition to chronic neuropathic pain being associated with altered thalamic burst firing and altered thalamocortical rhythm (Di Pietro et al., 2018; Jones, 2010; Sarnthein, Stern, Aufenberg, Rousson, & Jeanmonod, 2006; Walton, Dubois, & Llinas, 2010; Walton & Llinás, 2010), we have recently shown that neuropathic pain in the orofacial region is characterised by significantly increased ISOs (<0.1 Hz) along the ascending pain pathway including at the level of the primary afferent synapse, thalamus and S1 contralateral to the ongoing pain (Alshelh et al., 2016). Curiously, the increases in infra‐slow oscillation along the pain pathway occur at approximately the same frequency as calcium waves in astrocytes, that is, 0.03–0.06 Hz, and it is known that astrocytes can modulate synaptic activity by releasing gliotransmitters (Crunelli et al., 2002). In an animal model of CRPS, spinal astrocytes are chronically activated at 4 weeks after tibial fracture and contributed to the maintenance of hind paw allodynia and reduced weight bearing (Li et al., 2015). Additionally, a human postmortem study found that in an individual with long‐standing CRPS, chronic astrocyte activation occurred in the spinal dorsal horn, most prominently at the level of the initiating injury (Del Valle, Schwartzman, & Alexander, 2009). Treatments inhibiting glial activation and its potential effects on neural activity may prove to be a promising avenue for an adequate treatment for CRPS.

Whilst we have shown altered resting rhythm in individuals with orofacial neuropathic pain, to date no one has reported if a similar phenomenon occurs in individuals with CRPS. The aim of this study was to use resting state functional magnetic resonance imaging (fMRI) to determine if, similar to orofacial neuropathic pain, CRPS is characterised by increased infra‐slow oscillation (i.e., increased power) in the ascending pain pathway. While we were not able to image the spinal cord dorsal horn adequately for the purposes of the current study, we can focus on the thalamus and S1, and we hypothesise that CRPS subjects will display increased ISOs in these two regions contralateral to the pain. Furthermore, we hypothesise that resting functional connectivity between the thalamus and S1 will be significantly stronger in CRPS subjects compared with controls and that this strength will be correlated with the intensity of ongoing pain.

## 2 MATERIALS AND METHODS

Fifteen subjects with upper‐limb CRPS (11 females, mean [±*SEM*] age: 47.5 ± 3.2 years) and 30 age‐ and sex‐matched pain‐free healthy controls (20 females, mean [±*SEM*] age: 44.2 ± 2.6 years) were recruited for the study. Each CRPS subject was age‐ and sex‐ matched to 2 healthy control subjects. CRPS subjects were diagnosed according to the International Association for the Study of Pain ‘Budapest’ diagnostic criteria (Harden, Bruehl, Stanton‐Hicks, & Wilson, 2007) and had ongoing pain for at least 3 months. They were eligible for the study if they reported pain in more than one body region, but their CRPS had to be their primary complaint and had to be in the upper limb. Handedness was assessed using the Edinburgh Handedness Inventory (Oldfield, 1971). Subjects were excluded if they did not satisfy standard MRI safety criteria and healthy control subjects were excluded if they suffered from any chronic pain. Informed written consent was obtained for all procedures, which were conducted under the approval by local Institutional Human Research Ethics Committees and consistent with the Declaration of Helsinki.

For the CRPS subjects, hyperalgesia was assessed via pinprick on the dorsal web space of the hand and allodynia with light brush strokes on the dorsum of the hands/forearms. Vasomotor signs of skin temperature asymmetry were assessed through touch, skin colour changes/asymmetry through visual observation, and sudomotor/oedema signs of sweating and oedema via touch and the use of a tape measure respectively. Visual observation of finger, hand and wrist movement was used to assess motor signs. Trophic changes to hair, nail and skin were visually assessed. CRPS subjects marked the intensity of their ongoing pain on a visual analogue scale (VAS) (0 = no pain to 10 = worst pain imaginable) three times a day for seven consecutive days during the week of the scanning session. The average of these 21 pain intensity ratings was taken as ‘diary pain’ intensity. Subjects also outlined the area of their ongoing pain on a standard drawing of the body and assessed their pain on the day of the scanning session on a 10 cm VAS, that is, ‘scan pain’ intensity.

In all 15 CRPS and in 19 of the control subjects several questionnaires assessing limb function and perception were also collected. These were the patient rated wrist/hand evaluation questionnaire which assesses pain in the wrist joint and functional difficulties of the wrist and hand (MacDermid, Turgeon, Richards, Beadle, & Roth, 1998), the foreignness of limb feelings questionnaire which assesses how one perceives their own limb (Galer & Jensen, 1999b), the Bath CRPS body perception disturbance scale which assesses self‐perception of the affected limb (Lewis & McCabe, 2010), and the QuickDASH questionnaire which assesses CRPS symptoms and the individual's ability to perform related activities (Hudak, Amadio, & Bombardier, 1996). For all four of these questionnaires, higher scores indicate greater pain and/or disability.

### 2.1 Tactile acuity

In addition to questionnaires assessing limb function, we measured tactile acuity with a two‐point discrimination (TPD) tool immediately following the MRI scanning session in the same 15 CRPS and 19 control subjects. Subjects rested their hand in a supinated position and kept their eyes closed. A TPD wheel (Exacta™, CA) was applied to the surface of the distal pad of the index finger with distances of 0, 2, 3, 4 and 5 mm between the two points. Each of the five distances was presented seven times in a pseudo‐randomised order, resulting in a total of 35 trials for each hand. The subjects reported if they felt one point or two points touching the skin. When the subject could not feel two points at 5 mm spacing, then the distance was gradually increased until two points could be felt. The percentage of two‐point perception was plotted against the distance between the points and fitted by a binary logistic regression (IBM SPSS Statistics for Windows, Version 24.0. Armonk, NY: IBM Corp.), resulting in a psychometric function of absolute threshold. From the binary logistic regression fit, the threshold was determined as the distance at which chance level (50%) of correct two‐point perception was reached. Significant differences between the left and right hands in controls and between the painful and non(less)‐painful hand in CRPS subjects were determined using paired *t* tests (*p* < .05, two‐tailed).

### 2.2 MRI scans

Each subject was positioned supine onto the MRI scanner bed and placed into a 3 Tesla MRI scanner (Achieva TX, Philips Medical Systems, The Netherlands), with their head in a 32‐channel head coil to which padding was added to prevent head movement. With the subject relaxed and with their eyes closed, a series of 188 gradient echo echo‐planar functional MRI image volumes using blood oxygen level dependent contrast were collected. The first 8 image volumes were not recorded but were included to allow for global signal stabilisation, leaving 180 fMRI image volumes collected over a period of 6 min. Each image volume contained 35 axial slices covering the entire brain (field of view = 240 × 240 mm, matrix size = 80 × 78, slice thickness = 4 mm, repetition time = 2,000 ms; echo time = 30 ms, flip angle = 90°, raw voxel size 3 × 3 × 4mm). In each subject, a high‐resolution 3D T1‐weighted anatomical image set, covering the entire brain, was collected (ultrafast gradient echo sequence [turbo field echo]; field of view = 250 × 250 mm, matrix size = 288 × 288, slice thickness = 0.87 mm, repetition time = 5,600 ms; echo time = 2.5 ms, flip angle = 8°, raw voxel size 0.87 × 0.87 × 0.87 mm).

### 2.3 MRI analysis

Using SPM12 (Friston et al., 1995) and custom software, the fMRI image sets were realigned and movement parameters examined to ensure no subject displayed >1 mm volume‐to‐volume movement in the X, Y and Z planes and 0.05 rad in the pitch, roll and yaw directions. Cardiac (frequency band of 60–120 beats per minute +1 harmonic) and respiratory (frequency band of 8–25 breaths per minute +1 harmonic) noise was modelled and removed using the Dynamic Retrospective Filtering (DRIFTER) toolbox (Sarkka et al., 2012). Global signal drifts were then removed using the LMGS detrending method described by Macey and colleagues (Macey, Macey, Kumar, & Harper, 2004). Any fMRI signal pattern correlated with the movement parameters was removed, using a method similar to the nonlinear LMGS detrending method developed by Macey and colleagues (Macey et al., 2004). The fMRI images were then co‐registered to each subject's T1‐weighted anatomical image, the T1‐weighted image spatially normalised to the Montreal Neurological Institute (MNI) template and the normalisation parameters applied to the fMRI images. This process resulted in the fMRI images being resliced into 2 × 2 × 2mm voxels. The fMRI images were then spatially smoothed using a 6 mm full‐width at half maximum (FWHM) Gaussian filter. In 3 of the 15 CRPS subjects, pain was restricted to the left side of the body. Given the ascending pain pathways from the dorsal horn to the thalamus are primarily crossed, we reflected the T1 and resting state fMRI scans of these 3 CRPS subjects across the midline. As a consequence, for all 15 CRPS subjects in our analysis, the left side of the brain is contralateral to their ongoing pain.

#### 2.3.1 ISOs

**FIGURE 1:**
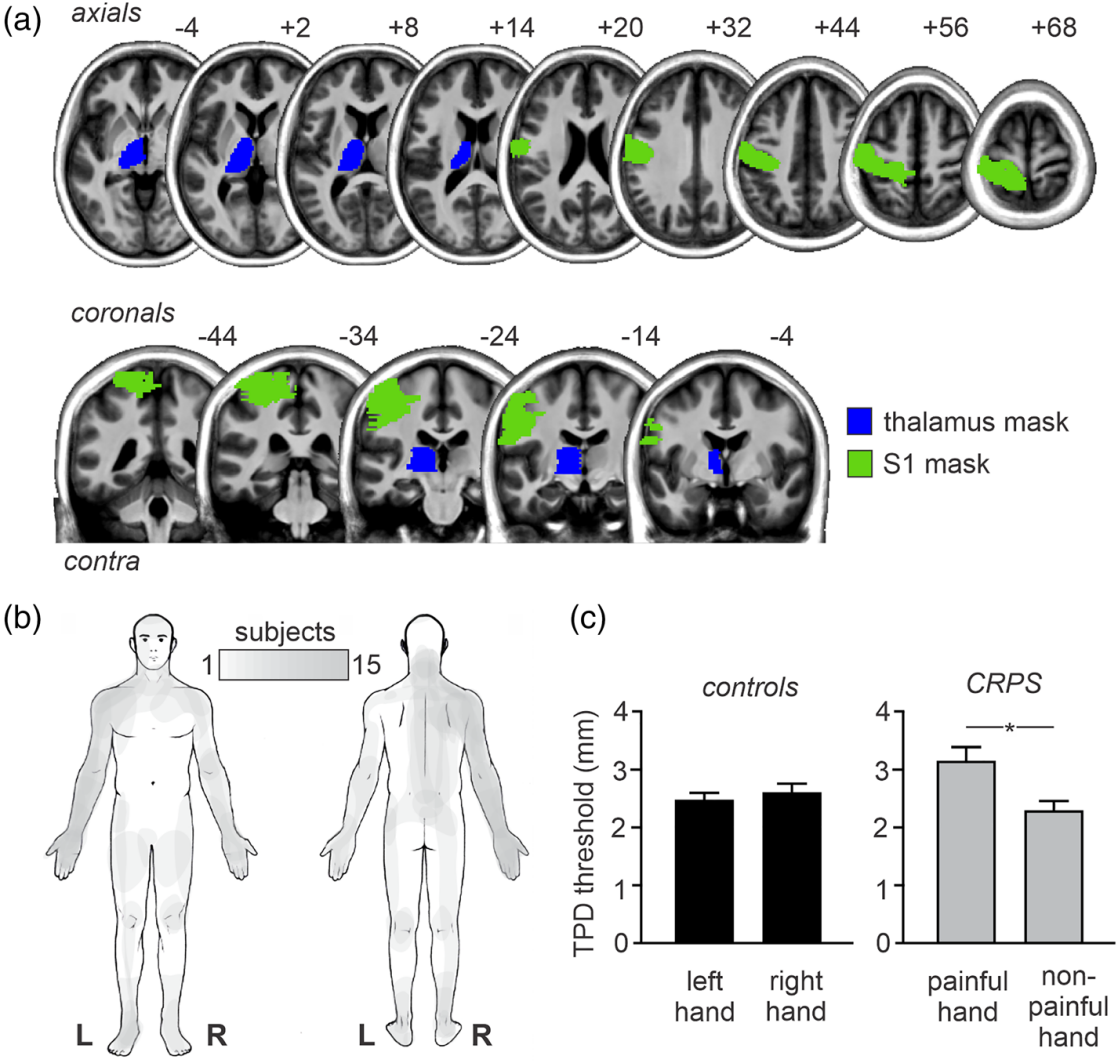
(a) Contralateral thalamus (blue) and primary somatosensory cortex (S1; green) masks used for restricted analysis overlaid onto a series of axial and coronal T1‐weighted images. The locations of slices in Montreal Neurological Institute Space are indicated at the top right of each axial and coronal slice. (b) Maps of ongoing pain in 15 subjects with complex regional pain syndrome (CRPS). Note that all 15 subjects have pain in the upper limb. (c) Two‐point discrimination thresholds (TPD) for the left and right hands in controls (black) and painful and non(less)‐painful hand in subjects with CRPS. Note that controls show similar values for the left and right hand whereas in CRPS subjects, the painful hand shows significantly greater TPD, that is, reduced tactile acuity, than the nonpainful hand. *p < .05

Using the SPM Data Processing Assistant for Resting‐State fMRI (DPARSF) toolbox (Chao‐Gan & Yu‐Feng, 2010), we calculated the sum of amplitudes of low‐frequency fluctuations (ALFF) between 0.03 and 0.06 Hz for each voxel in control and CRPS subjects using the spatially smoothed fMRI image sets. We also divided ALFF values by power over the entire frequency range to obtain fractional ALFF (fALFF) values for each voxel. Both ALFF and fALFF have high test–retest reliability, particularly ALFF (Zuo et al., 2010). In an initial analysis we determined significant differences between control and CRPS subjects over the entire brain at a voxel‐by‐voxel level using a two‐sample random effects procedure with age and sex as nuisance variables (*p* < 0.05, false discovery rate corrected for multiple comparisons). However, since our hypothesis was that CRPS subjects would display significant increases in 0.03–0.06 Hz power in the thalamus and S1 contralateral to the body area of the highest pain, we subsequently restricted our investigation to these brain regions by applying a mask (Figure 1a). Significant differences between groups were determined using a two‐sample random effects procedure with age and sex as nuisance variables (*p* < .05, false discovery rate corrected for multiple comparisons). For each significant cluster, fast Fourier transforms were performed on the resting fMRI signal intensity. More specifically, resting fMRI signals were demeaned, power spectral density calculated using MATLAB's ‘periodogram’ function, and the total power calculated as area under the curve with the ‘bandpower’ function. Plots of power at each frequency between 0.001 and 0.025 Hz were calculated and plotted for control and CRPS subjects. Total 0.03–0.06 Hz power was also calculated for each subject and plotted in addition to mean ± *SEM* of control and CRPS groups. We also determined if there were any significant differences between controls and CRPS subjects at other frequency ranges by calculating ALFF power for three standard ISO frequency domains: slow 5:0.01–0.027 Hz, slow 3:0.073–0.198 Hz, and slow 2:0.198–0.25 Hz. Significant differences between controls and CRPS subjects within the contralateral thalamus and S1 were then determined using two‐sample random effects procedures with age and sex as nuisance variables (*p* < .05, false discovery rate corrected for multiple comparisons).

To determine the potential effects of head movement on infra‐slow oscillation power, for each movement parameter (X, Y, Z, pitch, roll, yaw), in each subject, power spectra were calculated. The mean power between 0.03 and 0.06 Hz in control and CRPS groups was compared (*p* < .05, two‐tailed, two‐sample *t* test). The effects of pain variables were determined by assessing their relationships with 0.03–0.06 Hz power in significant clusters in CRPS subjects (Pearson's correlations, *p* < .05). Unfortunately, we were unable to test the effects of medication on infra‐slow power as all but 2 CRPS subjects were taking some form of medication.

#### 2.3.2 Regional homogeneity analysis

To assess local signal covariation, we measured Kendall's coefficient of concordance (KCC), which evaluates the similarity of the time series within each voxel and its nearest neighbours. Using the DPARSF toolbox, the fMRI preprocessed images were band‐pass filtered (0.03–0.06 Hz) and voxel‐based graphs generated for each individual subject. For each voxel, the KCC was computed from the time course of that voxel and its 19 neighbouring voxels. The analysis was restricted to the contralateral (left) thalamus and S1 by applying a mask. After smoothing the KCC maps using a 6 mm FWHM Gaussian filter, significant differences between groups were determined using a two‐sample random effects procedure with age and sex as nuisance variables (*p* < .05, false discovery rate corrected for multiple comparisons). We also extracted the KCC values from the thalamus cluster derived from the ALFF analysis and determined significant differences between controls and CRPS subjects using a two‐sample *t* test (*p* < .05, two‐tailed).

#### 2.3.3 Functional connectivity

ISO analysis revealed a cluster in the thalamus which displayed significant increases in ISO in CRPS subjects compared with controls. Using this cluster as a seed, we performed functional connectivity analyses to determine whether resting connectivity strengths between the thalamus and all other brain regions were significantly different in CRPS subjects compared with controls. For each subject, signal intensity changes over the 180 fMRI volumes were extracted from the thalamic seed and the relationship with ongoing signal intensities in each other voxel in the brain determined. The resulting thalamic connectivity maps were smoothed using a 6 mm FWHM Gaussian filter, and an initial analysis was performed in which we determined significant differences between control and CRPS subjects over the entire brain at a voxel‐by‐voxel level using a two‐sample random effects procedure with age and sex as nuisance variables (*p* < .05, false discovery rate corrected for multiple comparisons). Since our hypothesis was that CRPS subjects would display significant differences in thalamo‐S1 connectivity and that this relationship would be correlated to two‐point discrimination ability, we subsequently restricted our investigation to the contralateral S1 by applying a mask. Significant differences between groups were determined using a two‐sample random effects procedure with age and sex as nuisance variables (*p* < .05, false discovery rate corrected for multiple comparisons). For each significant cluster, thalamic connectivity values were extracted from each individual subject and the mean ± *SEM* values plotted.

Finally, we determined if the degree of connectivity between the thalamus and the S1 was related to ongoing pain intensity, pain duration or individual two‐point discrimination ability. Using the thalamic connectivity maps, voxel‐by‐voxel analyses were performed to determine significant linear relationships with either scan pain, diary pain or pain duration and the contralateral thalamus‐S1 connectivity (*p* < .05, false discovery rate corrected for multiple comparisons). The analysis was restricted to the contralateral S1 by applying a mask. In addition, we determined if the degree of thalamo‐S1 connectivity was correlated to two‐point discrimination of the painful hand in CRPS subjects and the right hand in control subjects. Two‐point discrimination measures were available in 12 CRPS and 19 control subjects. Using the thalamic connectivity maps, voxel‐by‐voxel analyses were performed to determine significant linear relationships with two‐point discrimination (*p* < .05, false discovery rate corrected for multiple comparisons). For each significant cluster, thalamic connectivity values were extracted from each individual subject and plotted.

## 3 RESULTS

The demographics and clinical characteristics of the 15 CRPS subjects are shown in Table 1 and the area of pain in all subjects is shown in Figure 1b. All 15 CRPS subjects reported ongoing pain in the upper limb and 7 also reported pain in the lower limb. Three CRPS subjects reported the highest ongoing pain in the left upper limb with the remaining reporting the highest pain in the right upper limb. Where subjects reported pain as bilateral in the upper limbs, the more painful side is termed the ‘painful’ side; the other as ‘non(less)painful’ from here on. The mean scan pain for CRPS subjects was 5.3 ± 0.5, mean diary pain was 4.6 ± 0.6 and the mean duration of pain was 4.7 ± 0.9 years. For the 3 CRPS subjects with pain on the left, their fMRI images were reflected across the midline so that the right side was ipsilateral to the highest ongoing upper limb pain.

**TABLE 1:** Demographics and clinical characteristics of patients with CRPS

Subject	Age	Sex	EHI score	Pain duration (years)	CRPS affected region^1^	Medications^3^	Pain intensity (diary VAS)	Pain intensity (scan VAS)
1	49	M	100.0 (R)	7.0	**R UL**, R LL, L LL, face, abdomen	None	4.5	4.0
2	56	F	100.0 (R)	4.2	**L UL**, R UL, R LL, R and L chest	Duloxetine, gabapentin, oxycodone, Quetiapine, Tapentadol	8.1	7.8
3	56	F	60.0 (R)	0.9	**R UL**, R neck, R chest	Ashwagandha, budesonide, cannabis, codeine, Formoterol, oxycodone, Paracetamol, salbutamol	8.3	7.9
4	62	F	100.0 (R)	6.2	**L UL**, *R UL* ^2^	Amitriptyline, Cannabidiol drops, codeine, levothyroxine, magnesium, Paracetamol, Topiramate, tramadol, valerian	5.8	4.3
5	58	F	−20.0 (A)	8.7	**R UL**, R LL, R face	Codeine, duloxetine, Linagliptin, meloxicam, metformin, Paracetamol	4.7	4.1
6	67	F	100.0 (R)	9.5	**R UL**, L UL, R LL, L LL	Amlodipine, gabapentin, ketamine in lipoderm cream, metformin, Metoprolol, pantoprazole, salbutamol	3.7	5.0
7	47	M	44.4 (R)	1.5	**R UL**	Amlodipine, atorvastatin, ibuprofen, Paracetamol, perindopril, Pregabalin	6.8	7.1
8	34	F	−23.1 (A)	5.3	**R UL**, R LL, R hip	Amitriptyline, buprenorphine patch	4.3	2.4
9	26	F	−40.0 (A)	1.3	**R UL**, L and R neck, Spine, L LL	None	5.4	6.8
10	46	F	80.0 (R)	3.9	**L UL**	Amitriptyline, Betahistine, duloxetine, naproxen, pantoprazole, Rizatriptan, Tapentadol, Valaciclovir,	0.6	5.6
11	24	F	70.0 (R)	2.6	**R UL**, L UL	Amitriptyline, gabapentin, levothyroxine	4.5	4.4
12	52	F	40.0 (A)	2.9	**R UL**, R torso	Ashwagandha, fish oil, ibuprofen, magnesium, mega B, melatonin, Paracetamol, Tapentadol, vitamin C, vitamin D	3.8	3.5
13	38	F	17.6 (A)	12.7	**R UL**, R neck, L LL	Duloxetine, gabapentin, naloxone, oxycodone, Palmitoylethanolamide (PEA)	0.0	5.9
14	52	M	−88.9 (L)	1.9	**R UL**, L and R neck, back	Cholecalciferol, ibuprofen, magnesium, oxycodone, Paracetamol, Pregabalin, tramadol, venlafaxine, Zopiclone	7.0	7.6
15	45	M	(L)	1.2	**R UL**		2.0	3.0

*Note:* **Bold** indicates the CRPS region with the most severe pain. *Italics* indicates remission of the CRPS region. Underline indicates medication taken in the last 24 hr of the day of testing.

Abbreviations: A, ambidextrous; L, left; LL, lower limb; R, right; *SEM*, standard error of mean; UL, upper limb.

#### 3.1.1. Questionnaires and two‐point discrimination threshold

Three of the 15 CRPS subjects were excluded from TPD analysis due to ongoing pain preventing testing, resulting in 12 CRPS and 19 control subjects. As hypothesised, in CRPS patients, the painful hand had a significantly greater TPD threshold, that is, reduced tactile acuity, than the non(less)‐painful hand (mean ± *SEM* TPD mm; painful: 3.16 ± 0.24, non(less): 2.30 ± 0.17; *p* = .001), whereas there was no significant difference between the left and right hands in controls (left: 2.47 ± 0.11, right: 2.60 ± 0.15; *p* = .21; Figure 1c). In addition to CRPS subjects having reduced tactile acuity in the painful hand, they also had significantly greater functional difficulties in their painful hand than controls as assessed by the Patient Rated Wrist/Hand Evaluation questionnaire (total mean ± *SEM* score: controls 3.7 ± 2.5, CRPS: 175.2 ± 21.7, *p* < .0001) and the QuickDASH questionnaire (disability/symptom mean ± *SEM* score: controls 1.9 ± 0.6, CRPS: 63.3 ± 4.8, *p* < .0001). As predicted CRPS subjects rated their limb as more foreign to themselves than controls as assessed by the foreignness of limb feelings questionnaire (total mean ± *SEM* score: controls 0.8 ± 0.8, CRPS: 48.3 ± 8.1, *p* < .0001) and had greater body perception disturbances than controls as assessed by the Bath CRPS Body perception disturbance scale (disability/symptom mean ± *SEM* score: controls 9.3 ± 1.5, CRPS: 22.5 ± 2.6, *p* < .0001). Finally, there were no significant relationships between TPD of the painful hand and either scan pain (*r* = −.42, *p* = .17), diary pain (*r* = .26, *p* = .42), or pain duration (*r* = .11, *p* = .73).

#### 3.1.2. Altered ISOs

**FIGURE 2:**
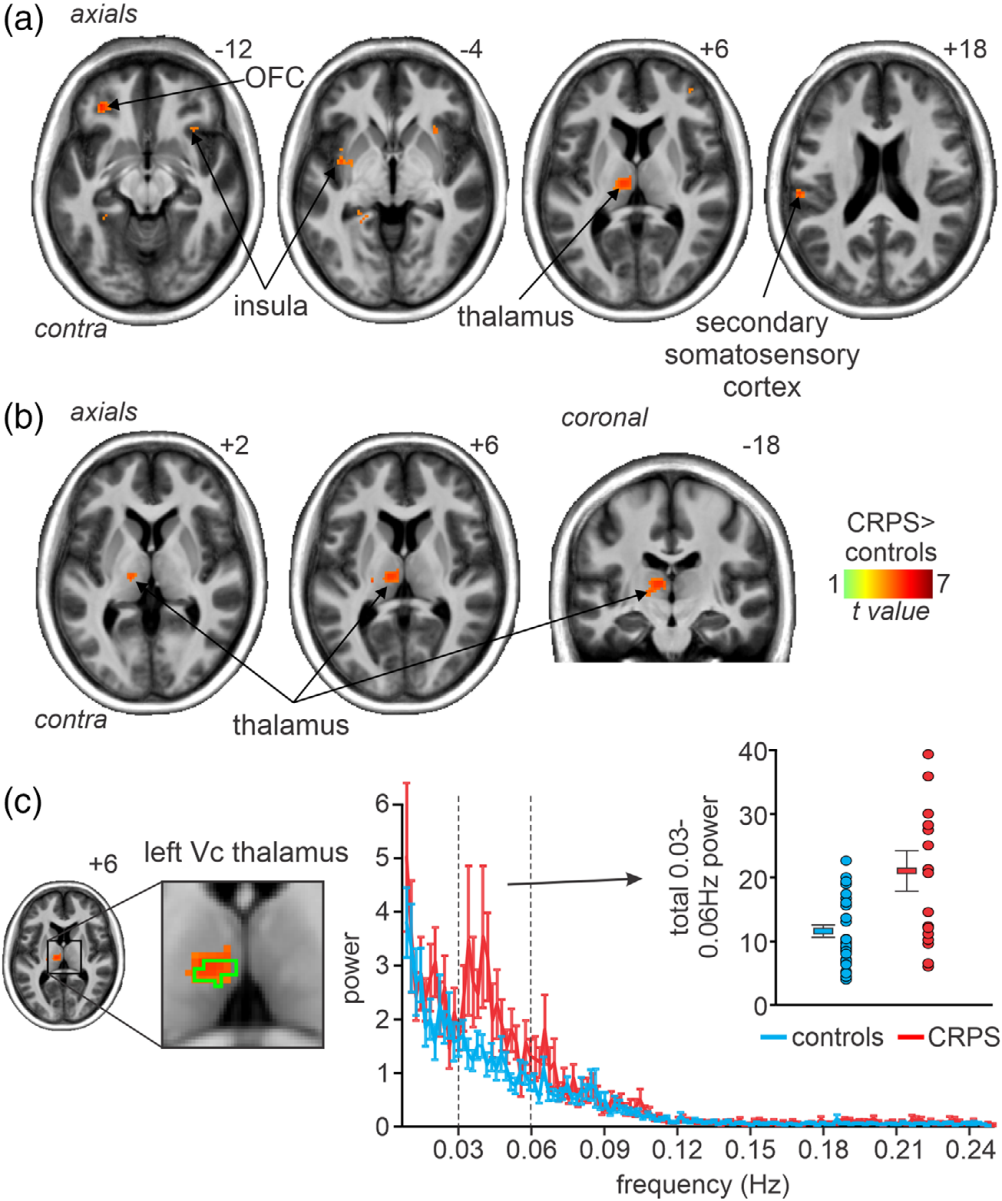
(a) Greater (*p* < .001, uncorrected) infra‐slow oscillation power (0.03–0.06 Hz; hot colour scale) assessed over the entire brain in 15 subjects with complex regional pain syndrome (CRPS) compared with 30 matched controls overlaid onto a mean T1‐weighted anatomical image set. The locations of slices in Montreal Neurological Institute Space are indicated at the top right of each axial slice. (b) Significantly greater (*p* < .05, FDR corrected) infra‐slow oscillation power (0.03–0.06 Hz; hot colour scale) assessed in the contralateral (to the highest pain) thalamus and primary somatosensory cortex. Slices locations in Montreal Neurological Institute Space are indicated at the top right of each slice. Note that the significant difference is in the region of the ventrocaudal (Vc) thalamus. (c) Plots of mean ± *SEM* power at each frequency between 0.01 and 0.25 Hz in controls (blue) and CRPS subjects (red). It is clear that the frequency band in which power was significantly greater in CRPS subjects was that between ~0.03 and 0.06 Hz. The green outline on the image slice to the left indicates the region activated by innocuous brushing of the hand and hence in the Vc thalamus. To the right are plots of individual subject and mean ± *SEM* 0.03–0.06 Hz total power for the thalamus cluster

Using a frequency range of 0.03–0.06 Hz, a voxel‐by‐voxel analysis of the entire brain revealed no significant group difference in either ALFF or fALFF values. However, for the ALFF values, when the statistical threshold was lowered to *p* < .001 uncorrected, a number of regions emerged. CRPS subjects had greater infra‐slow oscillation power in the contralateral orbitofrontal cortex, insula, thalamus and secondary somatosensory cortex and in the ipsilateral anterior insula (Figure 2a, Table 2). A restricted analysis of ALFF in the contralateral thalamus and S1 regions revealed that at *p* < .05 FDR corrected, CRPS subjects had significantly increased infra‐slow oscillation power in the contralateral thalamus (mean ± *SEM* 0.03–0.06 Hz ALFF power: *controls*: 1.01 ± 0.02, *CRPS*: 1.29 ± 0.05; Figure 2b, Table 2). This thalamus cluster overlapped with the region activated during innocuous brushing of the hand reported in our previous investigation (Wrigley et al., 2009), that is, in the region of the arm representation of the ventrocaudal (Vc) thalamus (Figure 2c). At no thalamus or S1 voxel was fALFF significantly different between controls and CRPS subjects. Extraction of power at each frequency in controls and CRPS subjects revealed that the frequency band showing significant power differences was remarkably restricted to between ~0.03 and 0.06 Hz (Figure 2c) and total power between 0.03 and 0.06 Hz was greater in CRPS compared with control subjects (mean ± *SEM* 0.03–0.06 Hz total power: *controls*: 11.7 ± 1.01, *CRPS*: 21.2 ± 3.3). The specificity of the power restriction to this particular band was confirmed by the findings that there were no significant ALFF power differences between control and CRPS subjects, in any thalamic region, in either slow 5 (0.01–0.027 Hz), slow 3 (0.073–0.198 Hz), or slow 2 (0.198–0.25 Hz) frequency bands.

**TABLE 2:** Montreal neurological institute (MNI) coordinates, cluster size and t‐score for regions of significant difference between control and CRPS subjects

Brain region	MNI co‐ordinate			Cluster size	t‐score
	x	y	z		
***Infra‐slow oscillation power (0.03–0.06 Hz)***					
*Wholebrain p < .001; CRPS > controls*					
Contralateral orbitofrontal cortex	−32	38	−14	39	4.97
Contralateral insula	−36	−2	−2	122	4.23
Contralateral secondary somatosensory cortex	−58	−26	20	71	5.34
Contralateral thalamus	−8	−18	8	92	4.58
Ipsilateral insula	30	20	−4	26	3.80
*Thalamus/S1 only p < .05 FDR; CRPS > controls*					
Contralateral thalamus	−8	−18	8	128	4.58
***Resting thalamic‐S1 connectivity***					
*Wholebrain p < .001; CRPS > controls*					
*CRPS > controls*					
Contralateral orbitofrontal cortex	−28	44	−8	53	4.64
Ipsilateral insula	34	18	0	50	4.21
Bilateral amygdala	−32	−4	−18	70	5.15
	32	−10	−14	42	4.08
Bilateral posterior parietal cortex	−54	−44	32	137	4.75
	58	−44	26	596	5.06 5.29
Cingulate cortex	−10	−46	44	1,737	
Bilateral S1	38	−20	38	52	4.54
	−46	−26	42	25	3.65
*S1 only p < .05 FDR; CRPS > controls*					
Contralateral S1	−20	−42	52	189	4.79
	−46	−26	42	37	3.56
*Increase thalamic‐S1 conn‐decrease scan pain*					
Contralateral S1	−26	−34	72	10	5.71
	−34	−44	72	19	5.35
*Increase thalamic‐S1 conn‐increase TPD*					
Contralateral S1	−60	−12	42	64	4.42
	−36	−26	66	13	3.63

In CRPS subjects, we found no significant relationship between thalamic 0.03 and 0.06 Hz power and scan pain (*r* = −.07, *p* = .79), diary pain (*r* = −.11, *p* = .67), or pain duration (*r* = −.09, *p* = .74). Furthermore, we found no significant relationship between thalamic 0.03 and 0.06 Hz power and Patient Rated Wrist/Hand Evaluation score (*r* = −.21, *p* = .48), QuickDASH score (r = −0.19, *p* = .50), Bath CRPS body perception disturbance scale (*r* = −.46, *p* = .08), or foreignness of limb feelings score (*r* = −.50, *p* = .06).

#### 3.1.3. Regional homogeneity

If increased ISO power results from increased synchronicity of astrocyte activation and recruitment of surrounding astrocytes and neurons, neighbouring voxels should display increased signal intensity synchronisation. We measured Kendall's coefficient of concordance (KCC) as an index of similarity of time series between voxels and found no significant differences between controls and CRPS subjects in the contralateral thalamus and S1. Furthermore, extraction of KCC values from the thalamic cluster derived from the ISOs analysis also revealed no significant difference in regional homogeneity in CRPS subjects compared with controls (mean ± *SEM* KCC *controls* 0.98 ± 0.01, *CRPS* 1.01 ± 0.01, *p* = .08; two‐sample *t* test).

#### 3.1.4. Thalamic‐somatosensory cortex connectivity

**FIGURE 3:**
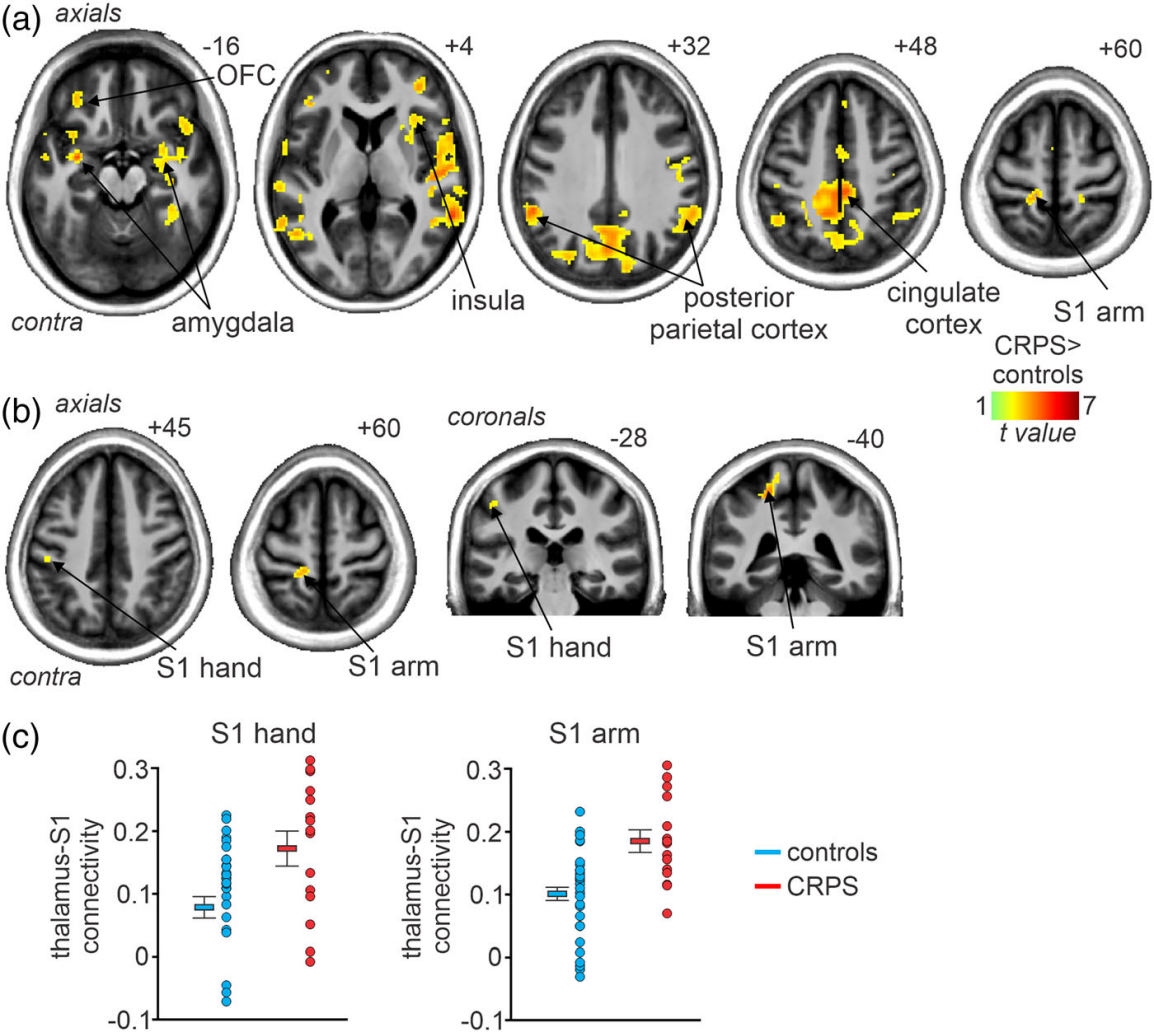
(a) Significantly greater (*p* < .05, FDR corrected; hot colour scale) resting functional connectivity between the thalamus and all other brain regions in 15 subjects with complex regional pain syndrome (CRPS) compared with 30 matched controls overlaid onto a mean T1‐weighted anatomical image set. Slices locations in Montreal Neurological Institute Space are indicated at the top right of each axial slice. (b) Significantly greater (*p* < .05, FDR corrected; hot colour scale) resting functional connectivity between the contralateral (to highest pain) thalamus and primary somatosensory cortex (S1). Slices locations in Montreal Neurological Institute Space are indicated at the top right of each slice. Note that the significant greater connectivity in CRPS subjects includes the regions receiving inputs from the arm/trunk and hand. (c) Plots of individual subject and mean ± *SEM* thalamic connectivity in the S1 region representing the arm and the S1 region representing the hand

Using the thalamic cluster derived from the infra‐slow oscillation analysis as a seed, we performed resting state functional connectivity between the contralateral thalamus and S1. A voxel‐by‐voxel analysis of the entire brain revealed greater thalamic connectivity in CRPS subjects compared with controls in a number of brain regions including the contralateral orbitofrontal cortex, ipsilateral insula, bilateral amygdala, bilateral posterior parietal, bilateral cingulate and bilateral S1 cortices (Figure 3a, Table 2). In no brain region was thalamic connectivity strength greater in controls compared with CRPS subjects. Restricted analysis of the contralateral S1 revealed two clusters in which CRPS subjects had significantly greater connectivity than controls. These clusters were in the locations that receive inputs from the arm (thalamic connectivity mean ± *SEM*: *controls* 0.10 ± 0.01, *CRPS* 0.19 ± 0.02) and the hand (*controls* 0.08 ± 0.02, *CRPS* 0.18 ± 0.03; Figure 3b,c, Table 2). In no voxel was thalamic connectivity strength greater in controls compared with CRPS subjects.

**FIGURE 4:**
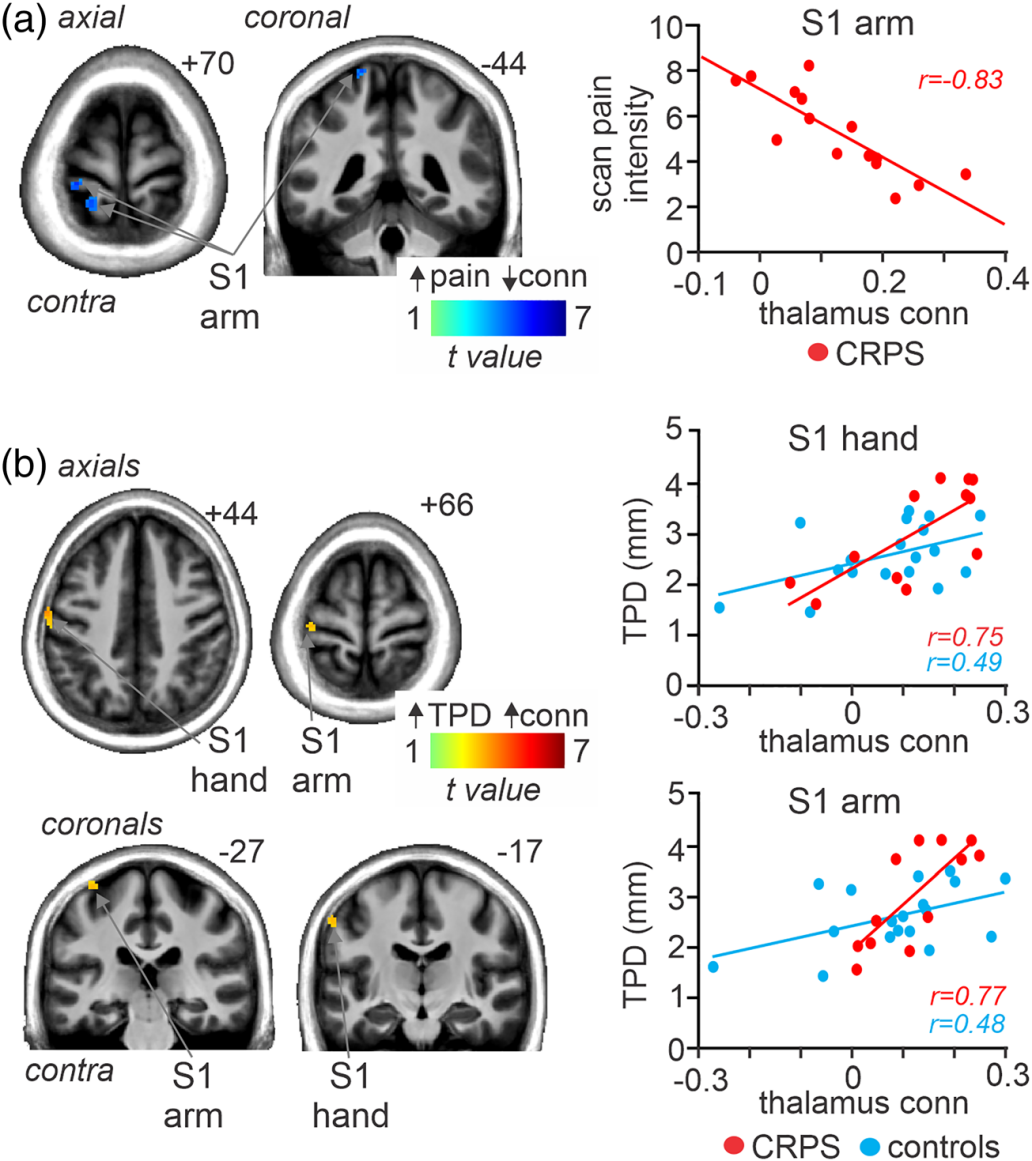
(a) Region of primary somatosensory cortex (S1) in which thalamic connectivity strength was significantly negatively (cool colour scale) correlated to scan pain intensity in 15 subjects with complex regional pain syndrome (CRPS). The locations of slices in Montreal Neurological Institute Space are indicated at the top right of each axial and coronal slice. To the right is a plot of individual subject connectivity strengths against scan pain intensity. (b) S1 regions in which connectivity strength was positively (hot colour scale) correlated to two‐point discrimination (TPD; in mm) threshold in 12 CRPS and 19 control subjects. To the right are plots for individual subject connectivity strength values against TPD for two S1 clusters, one in the S1 region representing the hand and another in the S1 region representing the arm. Values for these S1 clusters are plotted for CRPS and control subject separately. conn, connectivity

A voxel‐by‐voxel analysis of the contralateral S1 using scan pain intensity as a regressor resulted in a discrete region representing the arm where greater thalamic connectivity was associated with reduced pain (*r* = −.83; Figure 4a; Table 2). No significant correlation was found between thalamic connectivity and either diary pain or pain duration. In contrast, there was a significant positive relationship between thalamo‐S1 connectivity strength and TPD threshold in all subjects in two regions of the contralateral S1, one in the region representing the hand and another in the region representing the arm (Figure 4b; Table 2). These positive relationships were similar in controls and CRPS subjects, that is, the lower the tactile acuity the greater the connectivity strength in both the region of the arm (*controls r* = .48, *CRPS*, *r* = .77) and hand (*controls r* = .49, *CRPS*, *r* = .75).

## 4 DISCUSSION

Consistent with our hypothesis, we found increased infra‐slow oscillatory power in the contralateral thalamus of individuals with CRPS compared with controls. This thalamic increase overlapped with the region of the Vc thalamus and was restricted to the frequency band 0.03–0.06 Hz. Whilst we did not find a significant difference in infra‐slow oscillatory power in the contralateral primary somatosensory cortex, we did find that resting thalamo‐S1 connectivity strength was significantly greater in CRPS subjects compared with controls and was negatively correlated to scan pain intensity and tactile acuity. Finally, we found that regional homogeneity was not significantly different in the thalamus or S1 of CRPS subjects compared with controls.

We have previously shown that chronic orofacial neuropathic pain is associated with an increase in resting ISOs along the ascending pain pathway, including the Vc thalamus, thalamic reticular nucleus and S1 (Alshelh et al., 2016). In our previous study, we divided the thalamus contralateral to the side of pain into 240 regions of interest and found that the most common frequency band to display a significant infra‐slow oscillation difference was between 0.03 and 0.06 Hz. Whilst we did not perform the same regions of interest analysis in the current study, extraction of power over the entire frequency band (0.01–0.25 Hz) revealed that CRPS subjects also showed an increase in resting power that was restricted to the 0.03–0.06 Hz frequency band. Given that this frequency band is similar to that in which astrocyte calcium and gliotransmission release waves have been recorded (Scemes & Giaume, 2006) and given that there is preclinical and human postmortem evidence of chronic astrogliosis in individuals with CRPS and other neuropathic pain conditions (Del Valle et al., 2009; Li et al., 2015; Shi, Gelman, Lisinicchia, & Tang, 2012), we speculate that these oscillatory activity increases in CRPS result from chronic astrogliosis. It is known that in activated astrocytes, calcium waves can propagate to neighbouring astrocytes and this can result in neighbouring astrocytes responding with synchronous oscillatory gliotransmitter release (Cornell‐Bell, Finkbeiner, Cooper, & Smith, 1990; Scemes & Giaume, 2006). Such oscillatory gliotransmitter release can elicit long‐lasting NMDA‐mediated currents in surrounding neurons and recent evidence shows that astrocytes can elicit prolonged neural firing increases (Crunelli et al., 2002; Deemyad, Lüthi, & Spruston, 2018). It has been shown that increased ISOs are coupled to high frequency power fluctuations in the cortex (Mantini, Perrucci, Del Gratta, Romani, & Corbetta, 2007; Vanhatalo et al., 2004) and it is possible that the increases in ISOs within the thalamus of CRPS subjects lead to altered higher‐power activity in thalamocortical circuits. Altered thalamocortical loop dynamics is consistent with our finding of a significant increase in thalamo‐S1 connectivity strength in CRPS subjects, which may contribute to the thalamocortical dysrhythmia observed in CRPS and other neuropathic pain conditions (Di Pietro et al., 2018; Jones, 2010; Sarnthein et al., 2006; Walton et al., 2010; Walton & Llinás, 2010).

In our previous study in chronic orofacial neuropathic pain, we found that within the ascending pain pathway, only the primary afferent synapse, that is, the spinal trigeminal nucleus, displayed a significant increase in regional homogeneity (Alshelh et al., 2016). An increase in regional homogeneity reflects an increase in local synchrony of ISOs which would occur if infra‐slow frequency calcium waves propagate among neighbouring astrocytes. Whilst we were not able to explore the dorsal horn using fMRI in CRPS subjects in the current study, our finding that regional homogeneity was not different between CRPS and control subjects is consistent with the idea that chronic astrocyte activation at the primary afferent synapse is driving ISOs throughout the ascending pain pathway. Indeed, this idea is supported by an experimental animal investigation in which extracellular single‐unit neuronal activity was recorded in naïve rats and in those with chronic neuropathic pain induced by sciatic chronic constriction injury (Iwata et al., 2011). Firstly, the investigation reported spontaneous oscillations in ventroposterior thalamus neural firing at approximately 0.03 Hz in animals with neuropathic pain but not in naïve animals. Secondly, they found that severing the connection between the dorsal horn and thalamus eliminated these increases in infra‐slow neural activity oscillations, suggesting that they are being driven from the dorsal horn. We also recently found that in subjects with chronic orofacial neuropathic pain, administration of palmitoylethanolamide, a substance that can blunt astrocyte activation (Scuderi et al., 2012), significantly reduced ongoing pain intensity in 16 of 22 subjects (Alshelh et al., 2019). Whilst infra‐slow oscillation power in the spinal trigeminal nucleus was reduced in all subjects, it was only those subjects that also displayed reductions in thalamic ISOs that experienced pain relief. Furthermore, only those that displayed pain relief also showed reductions in connectivity between the spinal trigeminal nucleus and the thalamus. These results add further weight to the hypothesis that chronic neuropathic pain is driven by chronic astrocyte activation at the level of the primary afferent synapse.

In addition to increased infra‐slow oscillations, we found that connectivity strength between the thalamus and S1 was significantly correlated to pain and tactile acuity. Consistent with previous literature, in CRPS subjects, the painful hand displayed poor tactile acuity relative to the non(less)‐painful hand and pain‐free controls (Catley, O'Connell, Berryman, Ayhan, & Moseley, 2014; Lewis & Schweinhardt, 2012; Maihofner & DeCol, 2007; Pleger et al., 2006; Reiswich et al., 2012). We found, in both CRPS and control subjects, the strength of connectivity between the thalamus and S1 was positively correlated, that is, the poorer the tactile acuity the greater the connectivity strength. Similarly, despite no significant relationship between tactile acuity and ongoing pain intensity, scan pain scores were significantly negatively correlated to thalamo‐S1 connectivity in the region of S1 that receives information from the upper limb.

The direction of these correlations appears at odds with what one might expect, that is, since thalamo‐S1 connectivity is greater in CRPS subjects one might hypothesise that the greater the connectivity strength the greater the ongoing pain. Furthermore, it is surprising that greater thalamo‐S1 connectivity strength is associated with higher TPD thresholds, that is, reduced tactile acuity in control and CRPS subjects. It is known that in healthy controls, increased tactile discrimination ability evoked by short‐term co‐activation protocols results in expanded S1 representations (Pleger et al., 2001; Pleger et al., 2003). CRPS patients have been shown to have decreased perceptual learning ability with a tactile stimulation training program (Maihofner & DeCol, 2007) as well as smaller functional S1 representation of the affected hand in S1, consistent with impaired two‐point discrimination ability (Di Pietro et al., 2013). A recent investigation in pain‐free controls reported that better tactile acuity was associated with stronger connectivity in somatosensory discriminatory networks, although this study did not specifically explore Vc‐S1 connectivity strengths (Heba et al., 2017). Indeed, functional connectivity studies specifically targeting the Vc thalamus are rare and the interpretation of changes within this circuitry are difficult since Vc thalamus projections to S1 are complex. It is well‐established from tract tracing studies that Vc projections to S1 involve collaterals to GABAergic neurons of the thalamic reticular nucleus, which in turn projects back to the Vc thalamus and itself receives input from S1 (Pinault, 2004). It is thought that this circuitry controls thalamocortical rhythm (Fuentealba & Steriade, 2005) and we recently reported that thalamic GABA levels are strongly correlated to thalamocortical rhythm in chronic orofacial pain patients, despite there being no difference in thalamic GABA levels between pain patients and controls, and interestingly the strong correlation only seems to exist in the pain state (Di Pietro et al., 2018). We have also shown that thalamic GABA content is negatively correlated with Vc‐S1 connectivity with no significant relationship in pain‐free controls (Henderson et al., 2013). It is possible that the negative correlation between thalamo‐S1 connectivity and pain and the positive relationship with tactile acuity result from a combination of excitatory ascending and inhibitory descending controls which may also be responsible for the thalamocortical dysrhythmia in CRPS (Walton et al., 2010). These relationships will only be fully appreciated with a more complete understanding of the significance of functional connectivity and the interaction between various nuclei that regulate thalamocortical loop dynamics.

There are several limitations needing discussion. Firstly, we recruited and investigated a limited number of CRPS subjects, although given the relatively rare nature of this disorder 15 is a considerable number and is larger than most of the previous neuroimaging studies exploring CRPS and brain function (Di Pietro et al., 2013). Whilst one could argue that a whole brain, voxel‐by‐voxel analysis was underpowered, our spatially restricted analysis based on our a priori hypothesis revealed significant difference at corrected *p* values using population based statistical tests, and thus our study was adequately powered. Secondly, our investigation was cross‐sectional in nature and we did not assess individuals before they developed CRPS. Therefore, we cannot determine if any of the changes reported here occur throughout the development of CRPS or exist even before the initiating injury. A larger sample of subjects, recruited early in the course of the disorder and followed longitudinally, would allow us to explore changes that occur during the course of the condition. Thirdly, since we were interested in determining changes within the ascending pain pathway which is known to be lateralized, it was necessary to reflect the images of 3 CRPS subjects across the midline. Our results suggest that increased infra‐slow oscillation patterns are consistent with the contralateral projection of these pain pathways, although there remains a potential issue of lateralized effects. Finally, we were unable to determine the effects of medication on our results since 13 of the 15 CRPS subjects were taking daily medication for pain relief. Whilst the use of medications almost certainly affects aspects of the changes reported in this study, it has been shown that pain medications such as gabapentin, which was used by many of the CRPS subjects in this study, reduces astrocyte calcium signalling (Tian et al., 2005), thus we may have underestimated the difference in infra‐slow oscillation power between CRPS subjects and controls. In any case, further studies in which CRPS subjects are not taking medication are needed to confirm our results, though this is difficult on a practical and ethical level. Despite these limitations, the discrete changes in ISOs in the thalamus and the alteration in thalamo‐S1 connectivity strengths are consistent with previous investigations in chronic pain and we are confident they are important in the maintenance of CRPS.

## 5 CONCLUSIONS

Our findings show that the thalamic region that processes somatosensory information displays significantly increased ISOs in CRPS. Furthermore, CRPS is associated with increased thalamo‐S1 connectivity which is correlated with reduced tactile discrimination of the painful hand. These changes may underlie the well‐described alterations in thalamocortical loop dynamics, tactile discrimination and the constant perception of pain in individuals with CRPS.

## CONFLICT OF INTERESTS

The authors declare no potential conflict of interest

## Data Availability

Research data are not shared due to human ethics requirements.
